# Experimental Evaluation of a Recrosslinkable CO_2_-Resistant Micro-Sized Preformed Particle Gel for CO_2_ Sweep Efficiency Improvement in Reservoirs with Super-K Channels

**DOI:** 10.3390/gels10120765

**Published:** 2024-11-24

**Authors:** Adel Alotibi, Tao Song, Ali Al Brahim, Baojun Bai, Thomas Schuman

**Affiliations:** 1 Department of Earth Science and Engineering, Missouri University of Science and Technology, Rolla, MO 65409, USA; ajan75@mst.edu (A.A.);; 2 Department of Chemistry, Missouri University of Science and Technology, Rolla, MO 65409, USA; tschuman@mst.edu

**Keywords:** CO_2_-resistant particle gel, super-permeable channels, water alternating gas (WAG), enhanced oil recovery (EOR), conformance control, CO_2_-EOR, recrosslinkable preformed particle gel (RPPG)

## Abstract

A recrosslinkable CO_2_-resistant branched preformed particle gel (CO_2_-BRPPG) was developed for controlling CO_2_ injection conformance, particularly in reservoirs with super-permeable channels. Previous work focused on a millimeter-sized CO_2_-BRPPG in open fractures, but its performance in high-permeability channels with pore throat networks remained unexplored. This study used a sandpack model to evaluate a micro-sized CO_2_-BRPPG under varying conditions of salinity, gel concentration, and pH. At ambient conditions, the equilibrium swelling ratio (ESR) of the gel reached 76 times its original size. This ratio decreased with increasing salinity but remained stable at low pH values, demonstrating the gel’s resilience in acidic environments. Rheological tests revealed shear-thinning behavior, with gel strength improving as salinity increased (the storage modulus rose from 113 Pa in 1% NaCl to 145 Pa in 10% NaCl). Injectivity tests showed that lower gel concentrations reduced the injection pressure, offering flexibility in deep injection treatments. Gels with higher swelling ratios had lower injection pressures due to increased strength and reduced deformability. The gel maintained stable plugging performance during two water-alternating-CO_2_ cycles, but a decline was observed in the third cycle. It also demonstrated a high CO_2_ breakthrough pressure of 177 psi in high salinity conditions (10% NaCl). The permeability reduction for water and CO_2_ was influenced by gel concentration and salinity, with higher salinity increasing the permeability reduction and higher gel concentrations decreasing it. These findings underscore the effectiveness of the CO_2_-BRPPG in improving CO_2_ sweep efficiency and managing CO_2_ sequestration in reservoirs with high permeability.

## 1. Introduction

CO_2_ flooding is one of the most applied and fastest-growing enhanced oil recovery processes. According to a survey conducted by Advanced Resources International, between 2010 and 2020, CO_2_-enhanced oil recovery (CO_2_-EOR) in the United States saw significant growth. In 2010, the incremental oil recovery from CO_2_-EOR was approximately 280,000 barrels per day (BOPD). By 2020, this number had increased to around 300,000 BOPD, representing a substantial rise over the decade. Furthermore, CO_2_-EOR and sequestration (CCS) are significant due to global warming, increasing calls for CO_2_ emission reductions and environmental protection. By injecting CO_2_ into oil reservoirs, CO_2_ helps improve oil recovery while simultaneously trapping CO_2_ underground [1,2,3].

The main mechanism of CO_2_ in reservoirs during enhanced oil recovery involves injecting CO_2_ to reduce the oil’s viscosity and interfacial tension, improving its flow pattern toward the production wells. This process works best above the minimum miscibility pressure (MMP), where CO_2_ and oil become fully miscible, enhancing the extraction of hydrocarbons. CO_2_ injection causes the oil to swell and reduces residual oil saturation, significantly boosting oil recovery rates [4]. However, the unfavorable mobility ratio between oil and gas (due to the high difference in density) and reservoir heterogeneity (void space conduits, natural fractures, high-permeability streaks) can cause an undesired and unpredicted preferential flow of CO_2_ and, therefore, early gas breakthrough and excessive gas production at a late stage [3,5,6,7].

Several techniques and chemical treatments have been proposed to mitigate the conformance and mobility issues with CO_2_ flooding, including water-alternating-CO_2_ slug injection to reduce viscous fingering and improve the microscopic efficiency due to the lower remaining oil saturation [8,9], viscosified water-alternating-gas injection [10], foam-assisted CO_2_ injection to prevent gravity and viscous instabilities [11,12], direct thickening of CO_2_ using chemical agents, such as polymers or surfactants, to increase the viscosity of the CO_2_ [13,14], and polymer gel treatments, including in situ gels, particle gels, and recrosslinkable preformed particle gel systems [5,15,16,17,18].

In situ polymer gels have been investigated and applied for CO_2_ conformance control. This method involves injecting gelants (polymer and crosslinker) to form a highly elastic semi-solid bulk gel within the reservoir to block/modify high-permeability channels and direct the CO_2_ flow toward lower-permeability zones. Polyacrylamide (PAM) and partially hydrolyzed polyacrylamide (HPAM), crosslinked with heavy metal ions such as chromium (Cr^3+^, Zr^4+^, Al^3+^, etc.), are the most prevalent gel treatments used in the oil industry [15]. Several studies have tested and evaluated different in situ polymer gel systems under CO_2_ conditions. For instance, Martin and Kovarik [19] investigated the efficacy of polymer gels in modifying CO_2_ profiles for enhanced oil recovery. They compared commercial gelant systems, identifying that phenolic and vinyl gels (maintaining greater than an 80% reduction in CO_2_ permeability) were more stable and effective in high-permeability zones compared with xanthan and polyacrylamide gels. Seright [16] evaluated five types of in situ gels (weak and strong resorcinol–formaldehyde gels, a Cr (III)–xanthan gel, a Cr (III)–acetate–HPAM gel, and a colloidal-silica gel). Coreflood experiments at pressures up to 1500 psi and a temperature of 41 °C demonstrated that all gels could significantly reduce the water permeability, though their effectiveness varied with repeated water-alternating-gas cycles. The gels generally showed greater stability and higher residual resistance factors (F_rr_) during initial brine injections compared with CO_2_ injections. Raje [20] discussed the development and testing of three in situ gel systems used to control the mobility of CO_2_ in matrix rock. The study primarily addresses the early breakthrough of CO_2_ due to unfavorable mobility ratios in heterogeneous reservoirs. Two systems are based on a new biopolymer, KUSP1, which gels when the pH is lowered, and the third system uses the reaction of sulfomethylated resorcinol and formaldehyde (SMRF) to form a gel. Laboratory experiments on Berea sandstone cores demonstrated significant permeability reductions (80–99%) for brine and CO_2_. Hou and Yue [21] developed a composite gel to combat CO_2_ channeling in enhanced oil recovery. The gel, made from sodium silicate and an organic polymer, proved highly effective with a 2% acrylamide monomer concentration and 5% sodium silicate. Despite the promising results of conventional (HPAM/Cr^3+^) in situ polymer gels in CO_2_ conformance control applications, Sun’s study [22] highlighted a significant concern: some gels can experience severe dehydration under various CO_2_ conditions, which raises questions about their reliability and effectiveness in such environments.

Preformed particle gel (PPG) was developed to address the limitations of conventional in situ gel systems in oil fields, such as unpredicted gelation times, uncontrolled gel properties, and limitations in treating large reservoir fissures. PPG is a superabsorbent particle gel that swells several times upon mixing with water, providing better control over gel properties. It selectively blocks high-permeability zones, improving sweep efficiency, particularly in mature fields with excessive water and gas production [23,24]. Furthermore, several PPGs have been developed specifically for CO_2_ applications, and they can be divided into CO_2_-resistant and CO_2_-stimuli/responsive gels. Sun [3] evaluated a novel CO_2_-resistant particle gel (CRG) that addresses the dehydration issues of traditional polyacrylamide-based PPGs in CO_2_ flooding. CRG maintains its structure and increases swelling in low pH conditions, effectively reducing the CO_2_ and water permeability in fractured sandstone by forming internal and external gel cakes. Zhao [25] developed a dual-network CO_2_-responsive preformed particle gel (DN-CRPPG) to mitigate CO_2_ channeling and leakage. The gel achieved a swelling ratio of 1.25 under 10 MPa CO_2_, reduced permeability with an Frr of 4.7 × 10^6^, and improved oil recovery by 32.6% during CO_2_ flooding, with an additional 14.9% boost via WAG flooding. It exhibited less than 15% performance loss after multiple cycles and demonstrated excellent stability under supercritical CO_2_ conditions.

**Figure 1:**
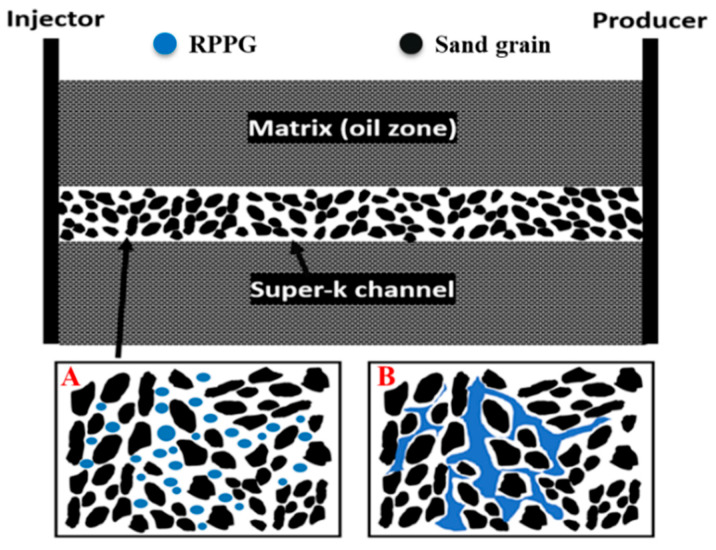
(**A**) A micro-size RPPG is injected to fill the pore space within the high-permeability channel. (**B**) RPPG is associated with forming a bulk, continuous-phase gel.

In extremely heterogeneous reservoirs, PPG faces limitations in effective plugging and can easily be washed out from the formation after water breakthrough [26]. To address these issues, recrosslinkable preformed particle gel (RPPG) was developed. The concept of this gel involves injecting a particle gel that swells in brine. Once in place, these particles can recrosslink to form a bulk gel due to their self-healing ability, making it more stable in larger reservoir fissures (Figure 1). Al Brahim [15] evaluated acrylamide-based RPPGs for improving conformance in natural gas (CH_4_) injection. The study demonstrates that RPPGs maintain their stability and strength under various conditions, including high-pressure gas exposure and acidic environments.

In this work, a novel CO_2_-resistant hyper-branched self-healing PPG (CO_2_-BRPPG) for CO_2_ conformance control was evaluated. We investigated basic gel characteristics such as swelling ratio, particle size distribution, gel rheology, and morphology at different pH and salinity values. The gel phase stability was evaluated under CO_2_ conditions using high-pressure vessels. Additionally, the gel plugging performance was systematically investigated in a WAG injection schema. The primary objective of this study was to develop a systematic evaluation method for assessing the stability and effectiveness of the CO_2_-BRPPG under brine/CO_2_ conditions during WAG cycles.

## 2. Results and Discussion

**Figure 2:**
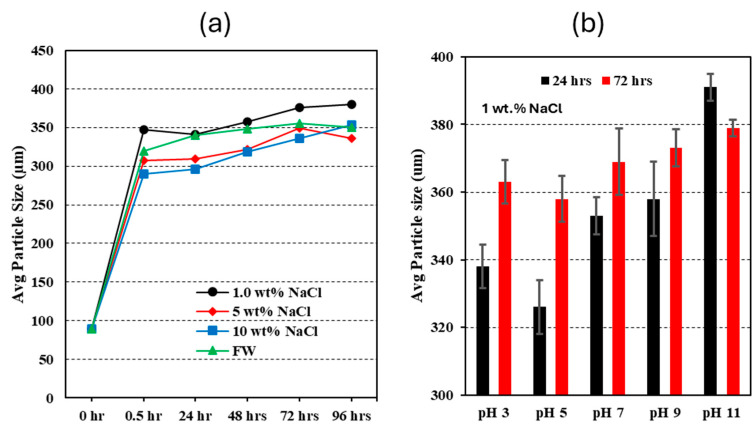
(**a**) Effect of salinity on particle size. (**b**) Effect of pH on particle size.

**Particle size and swelling ratio.** Due to the harsh conditions in the reservoir, including varying salinity levels and changes in the reservoir fluid pH caused by CO_2_ flooding, the properties of the CO_2_-BRPPG suspension may be affected. Therefore, evaluating the equilibrium swelling ratio of the CO_2_-BRPPG is essential for determining its ability to plug high-permeability channels effectively. The equilibrium swelling ratio (ESR) is defined as the time at which no further increase in swelling is observed. Generally, the ESR is determined by the balance between the gel’s elastic and osmotic pressure [27]. In our paper, we calculated the swelling ratio by measuring the particle size and applying Equation (1). The effect of the salinity and pH of the brine on the particle gel is shown in Figure 2 and Table 1.

In Figure 2a, the results show that, during the first 30 min, the swelling of the gel particles was rapid, achieving over 90% of their maximum size. This rapid swelling is attributed to the small size of the gel particles, which have a large surface area, increasing the likelihood of fluid exchange with the surrounding environment [28]. After this initial phase, the particle size increase slowed until no significant increase in the particle size was observed (ESR). Additionally, as the salinity increases, the ESR decreases. For example, a suspension with 1% NaCl increased the particle size 4.2 times (=76 ESR), while a suspension with 10% NaCl only increased the particle size 3.9 times (=61 ESR). This effect is due to the negative charges of the carboxylate (COO–) in the polymer chain causing electrostatic repulsion, expanding the polymer network, and increasing water accommodation. In brine solutions, cations reduce this repulsion between polymer chains by neutralizing negative charges and compressing the electrical double layer, decreasing the network expansion and water space. Thus, the swelling ratio of the CO_2_-BRPPG suspension decreases with higher brine salinity [29]. Similarly, in our sample test using formation water, the particle gel experienced a low ESR.

On the other hand, the effect of pH on the swelling ratio was evaluated using a particle gel prepared in 1 wt.% NaCl, as illustrated in Figure 2b. The graph illustrates the average particle size of the CO_2_-BRPPG across different pH levels, showing distinct variations with changes in pH. The changes in particle size at pH 3, 5, and 7 are small and comparable, with sizes of approximately 338 µm, 326 µm, and 353 µm, respectively. However, at pH 11, there is a significant increase in particle size to around 391 µm. These observations indicate that, as the pH changes, the particle size decreases at lower pH levels and increases significantly at higher pH levels. This phenomenon can be attributed to changes in the ionization state of the functional groups within the polymer chain. At lower pH levels, protonation of acidic groups (e.g., carboxyl groups) reduces the electrostatic repulsion, decreasing swelling. Conversely, at higher pH levels, deprotonation occurs [30]. However, the presence of NaCl screens the electrostatic repulsion between negatively charged groups, resulting in a reduced swelling ratio compared with what might be expected in the absence of salt [31]. This highlights the complex interplay between pH and ionic strength in determining the swelling behavior of particle gels.

**Figure 3:**
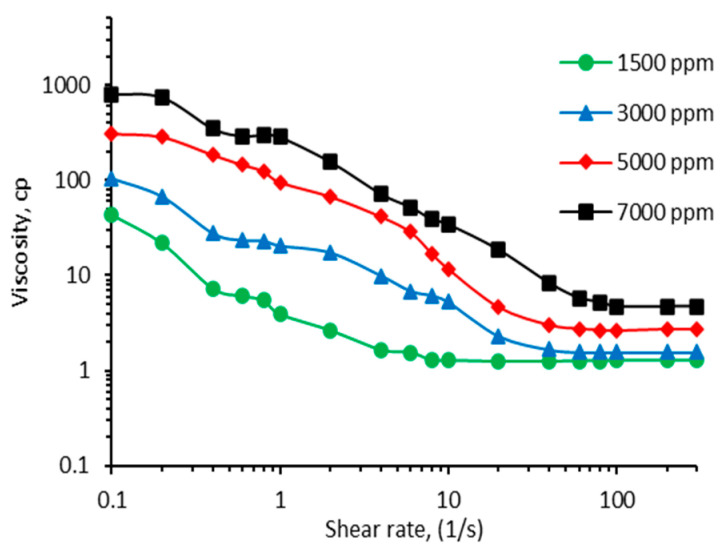
Viscosity as a function of the shear rate of the CO_2_-BRPPG suspension at different suspension concentrations.

**Rheology Characterization.** CO_2_-BRPPG is prepared as a suspension solution with a different concentration; therefore, evaluating its apparent viscosity is important. This property influences the gel’s flow behavior and propagation in the reservoir. The rheological behavior of the CO_2_-BRPPG was analyzed by examining the viscosity versus the shear rate at different concentrations (1500 ppm, 3000 ppm, 5000 ppm, and 7000 ppm), as shown in Figure 3. The results indicate a clear shear-thinning behavior, with viscosity decreasing as the shear rate increased for all concentrations. At lower concentrations (1500 ppm and 3000 ppm), the initial viscosities were lower, starting at around 40 CP and 100 CP, respectively, and decreased more significantly with increasing shear rates, ultimately converging toward lower viscosities at higher shear rates. In contrast, higher concentrations (5000 ppm and 7000 ppm) exhibited much higher initial viscosities, around 300 CP and 800 CP, respectively, which decreased more gradually, maintaining higher viscosities at elevated shear rates. This concentration-dependent viscosity behavior suggests that the gel’s resistance to flow increases with concentration, while the shear-thinning nature ensures that the gel can be easily injected under high shear conditions but retains sufficient viscosity under low shear conditions to block pathways effectively.

**Figure 4:**
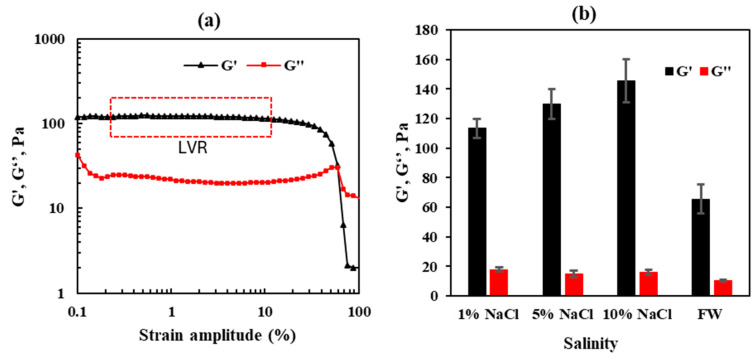
(**a**) The linear viscoelastic region of the CO_2_-BRPPG demonstrates consistent modulus values within this range. (**b**) The effect of salinity on gel strength.

CO_2_ is a highly diffusive gas with small, linear molecules that can infiltrate the gel matrix. This makes the gel more flexible and less mechanically robust, increasing the likelihood of damage under stress. Without sufficient strength, the gel is vulnerable to mechanical damage from CO_2_ penetration, reducing its effectiveness in blocking or diverting reservoir flow. We examined the impact of salinity on the gel strength. Figure 4a illustrates the storage modulus (G’) and loss modulus (G”) as functions of strain amplitude (%), providing insights into the gel’s behavior in the linear viscoelastic region. The G’ remains relatively constant at around 100 Pa up to a strain amplitude of approximately 10%, indicating that the gel maintains its elastic properties under small deformations.

Figure 4b illustrates the influence of salinity on gel strength, showing a clear trend of increasing gel strength with higher NaCl concentrations. The storage and loss modulus were measured for gels prepared in solutions of varying NaCl concentrations (1%, 5%, and 10%) and in formation water with a total dissolved solid content of 57,800 ppm. The results indicate that the gel prepared in a 10% NaCl solution exhibits the highest storage modulus (145 Pa), compared with 130 Pa in 5% NaCl, 113 Pa in 1% NaCl, and 66 Pa in formation water. Similarly, the loss modulus shows a slight variation with salinity, with values of 16 Pa in 10% NaCl, 15 Pa in 5% NaCl, 18 Pa in 1% NaCl, and 11 Pa in formation water. These findings suggest that the gel’s mechanical strength improves significantly with increasing NaCl concentrations. Higher salinity decreases the swelling ratio of the gel by reducing its water absorption capacity [26,32]. This reduction in swelling results in a more compact and dense gel structure, contributing to its increased rigidity and mechanical strength under stress.

**Phase stability and morphology test**. In this evaluation, we investigated the stability of the CO_2_-BRPPG by evaluating the change in the swelling ratio of the CO_2_-BRPPG under different CO_2_ conditions by varying the salinity and CO_2_ pressure. The pressures used were 500 psi, 850 psi (dense phase), and 1200 psi (supercritical CO_2_), all at a constant temperature of 45 °C. The aging time was kept constant at three days for all experiments. Under normal conditions, as previously shown (Figure 2a), the swelling ratio of a gel prepared in 1% NaCl after three days is typically 370 µm (=71 SR).

**Figure 5:**
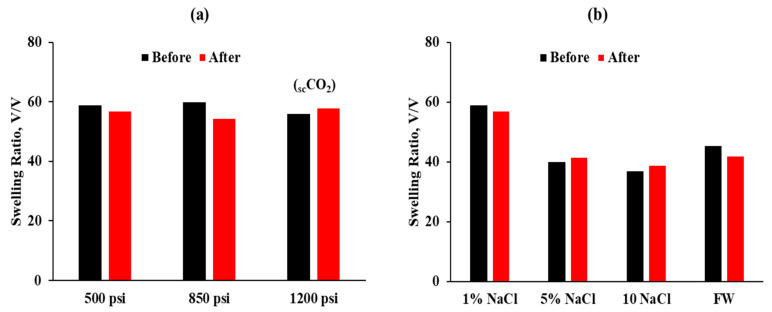
(**a**) Swelling ratio of the CO_2_-BRPPG before and after exposure to CO_2_ at pressures of 500 psi, 850 psi (dense phase), and 1200 psi (supercritical CO_2_). (**b**) Effect of salinity on the swelling ratio of the CO_2_-BRPPG under 850 psi CO_2_ pressure.

The results, depicted in Figure 5a, indicate no further increase in the swelling ratio under different CO_2_ pressures; instead, there is a slight decrease in the swelling ratio. Specifically, the swelling ratios after exposure to 500 psi, 850 psi, and 1200 psi CO_2_ remain relatively stable, with only minor decreases observed. Several interrelated factors can explain this observation. When CO_2_ dissolves in water in the particle gel suspension, it forms carbonic acid, which lowers the pH of the solution, making it more acidic. This decrease in pH can affect the ionization state of the functional groups within the gel, reducing the electrostatic repulsion and causing the gel network to contract [33]. High-pressure CO_2_ also has a high degree of solubility in water, increasing the concentration of CO_2_ molecules within the gel network [3]. The presence of these molecules can create additional osmotic pressure, counteracting the swelling pressure exerted by the absorbed water and leading to a slight reduction in the swelling ratio. Furthermore, CO_2_ is relatively hydrophobic compared with water, and high concentrations within the gel can promote hydrophobic interactions between polymer chains, resulting in a denser gel structure. Additionally, exposure to high-pressure CO_2_ might induce structural changes in the polymer network, such as partial crosslinking or densification, decreasing the gel’s ability to swell. These combined factors contribute to the observed reduction in the swelling ratio under high-pressure CO_2_ conditions [30,34]. Further studies are needed to isolate and understand the relative contributions of each mechanism to provide a comprehensive explanation.

**Figure 6:**
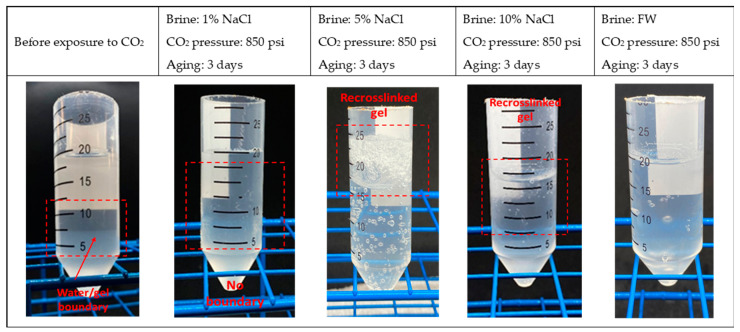
Set of pictures showing the CO_2_-BRPPG after exposure to CO_2_ at 850 psi for different salinities.

Figure 5b evaluates the effect of salinity under 850 psi CO_2_ pressure. Similar to the result in Figure 5a, the results show a slight decrease in the swelling ratio for 1% NaCl, 5% NaCl, and 10% NaCl solutions. Notably, the particle gel shows recrosslinking behavior at higher salinities, specifically at 5% and 10% NaCl, as shown in Figure 6. This recrosslinking behavior is evidenced by the reduction in the swelling ratio, indicating that part of the particle gel structure becomes more compact after exposure to CO_2_. Formation water samples also show a minor decrease in swelling ratio.

**Figure 7:**
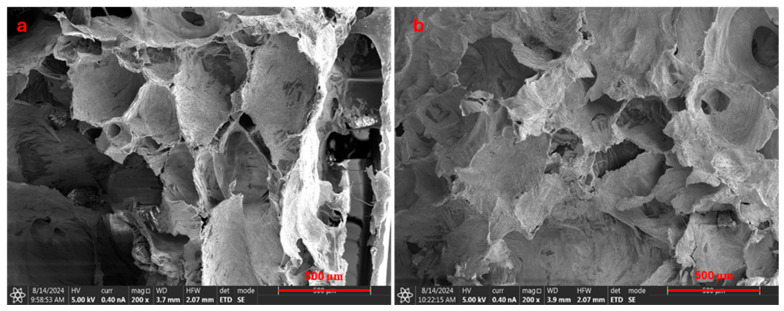
SEM picture for the gel before (**a**) and after (**b**) exposure to supercritical CO_2_ for 3 days.

To observe gel stability in the CO_2_ atmosphere, we carried out scanning electron microscopy analysis of the gel samples. Figure 7a,b shows the structural morphology of the gel samples before and after 3 days of exposure to supercritical CO_2_. Figure 7a shows the gel structure before CO_2_ exposure, which exhibits a highly porous network with large, interconnected voids and well-defined structural walls. The porous morphology suggests that the gel has a loosely packed structure. The voids appear relatively open and uncompressed, indicating the gel’s original, unaltered state. As shown in Figure 7b, after exposure to CO_2_, the gel structure appears denser and more compact. The pores seem smaller, and the structural walls appear more compressed in certain areas, suggesting a contraction or reorganization of the gel network.

**Figure 8:**
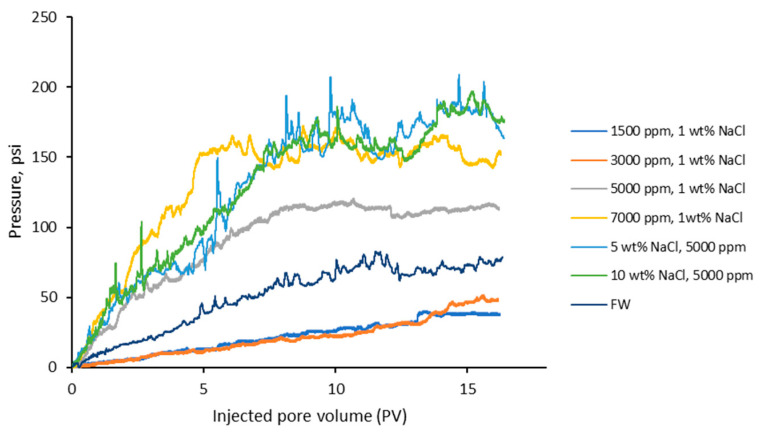
Profile of the injection pressure on the CO_2_-BRPPG microgel at different salinity and concentration values.

**Injectivity of the CO_2_-BRPPG.** The injection pressure behavior of particle gel suspensions at various concentrations and salinities as a function of injected pore volume (PV) is illustrated in Figure 8. All curves exhibit an initial increase in pressure, continuing until the gel starts to be produced from the outlet, followed by stabilization of the injection pressure. This trend indicates the balance between the retention of gel particles within the porous media and their transport through it. After the gel particles start to be produced from the sandpack, the injection pressure gradually stabilizes due to several key factors that balance the dynamics of gel retention and advection within the porous media. The formation of stable gel structures within the pore spaces partially blocks the pores, creating a consistent level of resistance to fluid flow. However, the gel requires time to reach this equilibrium, as the retention and transport rates of the gel particles adjust within the matrix. Once this balance is achieved, the overall resistance remains constant, leading to stable injection pressure.

**Figure 9:**
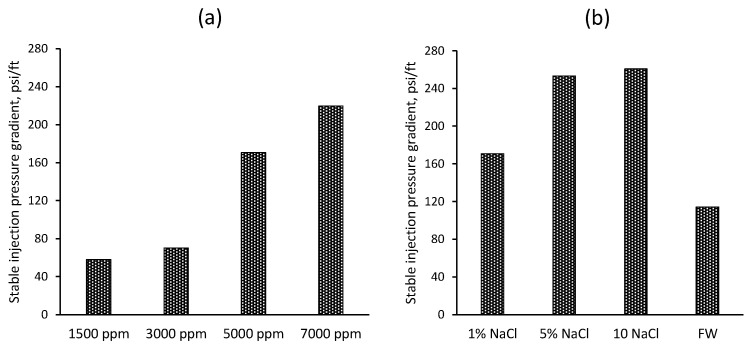
(**a**) Relationship of CO_2_-BRPPG concentration and stable injection pressure gradient. (**b**) The CO_2_-BRPPG suspension salinity and stable injection pressure gradient.

Figure 9a illustrates the stable injection pressure gradient (psi/ft) for gels at various concentrations (1500 ppm, 3000 ppm, 5000 ppm, and 7000 ppm). The stable injection pressure gradient increases with gel concentration, showing values of 58 psi/ft for 1500 ppm, 70 psi/ft for 3000 ppm, 171 psi/ft for 5000 ppm, and 220 psi/ft for 7000 ppm. This trend correlates with the previously observed gel viscosity results, where higher gel concentrations exhibited greater viscosities. As the gel concentration increases, the viscosity also increases, leading to more significant resistance to flow within the porous media. This higher viscosity results in a more substantial and stable injection pressure gradient, as the gel can better block and retain within the pore spaces.

On the other hand, Figure 9b illustrates the stable injection pressure gradient (psi/ft) for gels prepared in different salinity solutions (1% NaCl, 5% NaCl, 10% NaCl, and formation water). The stable injection pressure gradient values are 171 psi/ft for 1% NaCl, 253 psi/ft for 5% NaCl, 261 psi/ft for 10% NaCl, and 114 psi/ft for FW. These results correspond with the previously observed gel strength data, where higher salinity led to increased gel strength due to reduced swelling and a more compact gel structure. The gels prepared in higher-salinity solutions exhibit higher pressure gradients, indicating higher resistance to the gel advection. In contrast, the gel prepared in formation water shows a lower pressure gradient despite its high salinity, likely due to the complex mixture of salts used to prepare the brine.

Evaluating the resistance factor is crucial for optimizing gel treatments in field applications. It helps in understanding the transportation mechanism of the injected fluid within the reservoir, allowing for the design of effective gel formulations and injection strategies. In porous media, the resistance to flow is influenced by the fluid’s viscosity; therefore, the resistance factor can be seen as an indicator of the effective viscosity of the polymer gel. Table 2 shows the relationship between the CO_2_-BRPPG concentration and suspension salinity.

**CO_2_ flooding test.** After injecting the gel into the sandpack, the system was shut in for three days to allow the recrosslinking process to occur. CO_2_ flooding was then conducted in a WAG pattern. Initially, CO_2_ was injected in pressure steps to determine the breakthrough pressure through the treated matrix. Following this, three WAG cycles were performed to evaluate the gel’s plugging performance. In this experiment, we tested two variables: the effect of salinity and gel concentration on the plugging performance of the CO_2_-RPPG. As shown previously, increasing salinity enhances gel strength, which in turn increases the breakthrough pressure. Similarly, a higher gel concentration leads to greater retention within the matrix, thereby increasing the breakthrough pressure.

**Figure 10:**
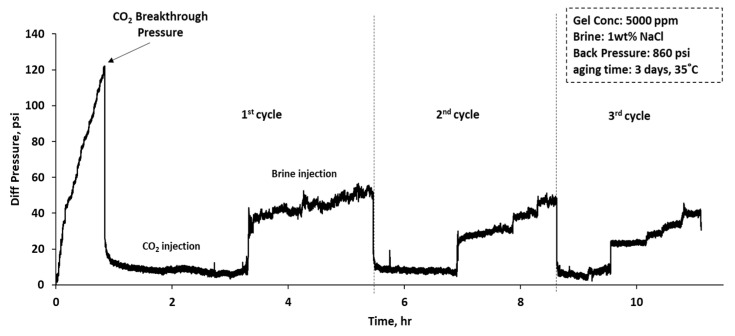
Differential pressure profile of water-alternating-CO_2_ injection in a sandpack treated with 3000 ppm CO_2_-BRPPG.

**Figure 11:**
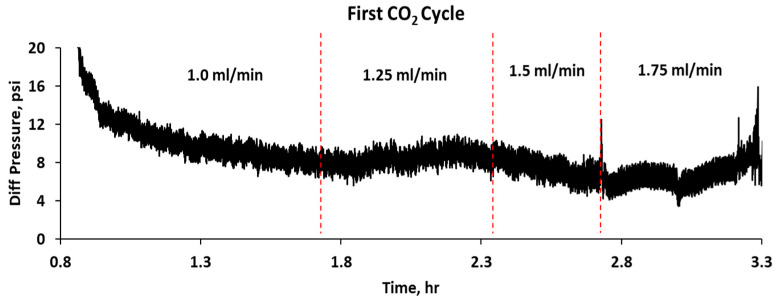
Differential pressure of the first CO_2_ injection cycle.

**Figure 12:**
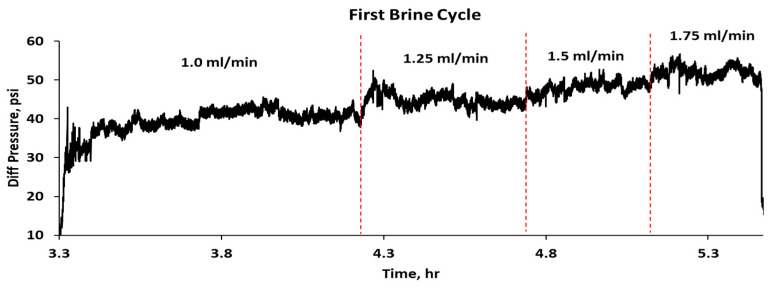
Differential pressure of the first brine injection cycle.

Figure 10 illustrates the differential pressure regarding time during a CO_2_–water injection experiment in a sandpack treated with 5000 ppm CO_2_-RPPG, encompassing three WAG cycles. Initially, CO_2_ is injected until it reaches a breakthrough pressure of approximately 121 psi, followed by a sharp pressure drop and stable flow with minor fluctuations, as shown in Figure 11. CO_2_ is then injected at four flow rates (1, 1.25, 1.5, and 1.75 mL/min), resulting in only small changes in pressure. Following that, brine injection at four flow rates, the same as the CO_2_ flow rates, results in stepwise increases in differential pressure, as shown in Figure 12. In the first WAG cycle, the pressure was lower compared with the second cycle for both CO_2_ and brine. It is hypothesized that the CO_2_ caused slight dehydration of the gel, leading to a decrease in pressure. Subsequently, brine injection resulted in the reswelling of the gel particles, as evidenced by the increase in the CO_2_ differential pressure during the second WAG cycle. CO_2_ can dehydrate polymer gel because it can diffuse into the gel matrix and dissolve the water present, leading to a reduction in the gel’s volume and its subsequent shrinkage [22]. Furthermore, under high-pressure conditions, CO_2_ in its supercritical or dense phase has a high affinity for water, which enhances its ability to extract water from the polymer gel, thus dehydrating it. The third cycle shows a drop in pressure differences compared with the first and second cycles.

**Figure 13:**
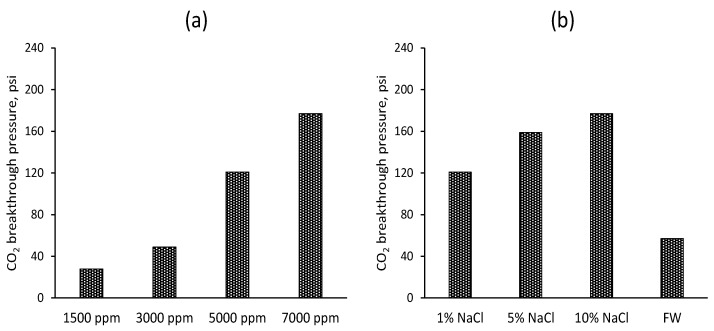
(**a**,**b**): CO_2_ breakthrough pressure for different gel concentrations and salinity conditions.

**Effect of the salinity and gel concentration of CO_2_ breakthrough pressure.** CO_2_ breakthrough pressure is defined as the pressure at which CO_2_ first overcomes the resistance provided by the gel and begins to flow through the porous media or fractures. Figure 13a illustrates the effect of gel concentration on CO_2_ breakthrough pressure, showing that, as gel concentration increases, the CO_2_ breakthrough pressure also rises. Specifically, at 1500 ppm, the CO_2_ breakthrough pressure is 28 psi; at 3000 ppm, it increases to 49 psi; at 5000 ppm, it further rises to 121 psi; and at 7000 ppm, it reaches the highest value of 177 psi. This trend can be correlated with data showing that higher gel concentrations lead to higher gel retention, which in turn increases the resistance against CO_2_ breakthrough. The increased gel retention enhances the plugging efficiency and viscosity of the gel, thereby impeding the flow of CO_2_ through the porous media or fractures and resulting in higher breakthrough pressures.

Figure 13b presents the effect of salinity on CO_2_ breakthrough pressure, showing that, as salinity increases, the CO_2_ breakthrough pressure also increases. Specifically, for 1% NaCl, the CO_2_ breakthrough pressure is 121 psi; for 5% NaCl, it increases to 159 psi; for 10% NaCl, it reaches the highest value of 177 psi; and for formation water, the CO_2_ breakthrough pressure is 57 psi. This trend indicates that higher salinity results in greater resistance to CO_2_ breakthrough. Recall that higher salinity is associated with higher gel strength. The increased salinity reduces the swelling ratio of the gel, making it more compact and robust.

**Figure 14:**
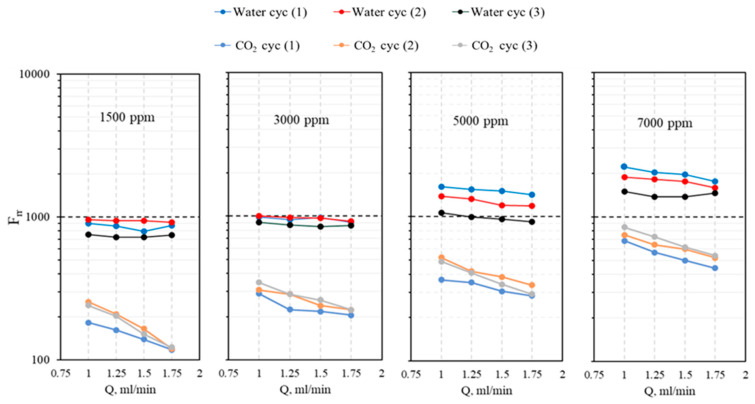
The residual resistance factor for both CO_2_ and water at different flow rates (1, 1.25, 1.5, and 1.75 mL/min) and gel concentrations (1500 ppm, 3000 ppm, 5000 ppm, and 7000 ppm).

**Effect of the gel concentration and salinity on the plugging performance.** Figure 14 illustrates the effect of salinity on the residual resistance factor for both CO_2_ and water when the gel suspension concentration is constant. It is noteworthy that the first CO_2_ injection cycle demonstrates lower plugging performance across all experiments. This phenomenon occurs because, during the initial CO_2_ injection, the system contains only gel. As CO_2_ breaks through and forms channels through the gel-treated matrix, the plugging performance diminishes. Subsequently, when brine is injected, these channels are filled with water, which adds extra resistance to the CO_2_ flow during the second CO_2_ injection. Consequently, the second CO_2_ injection cycle exhibits better plugging performance than the first cycle. Additionally, as the gel concentration increases, the F_rr_ for both gas and water increases. For gel concentrations of 1500 ppm and 3000 ppm, the plugging performance is comparably close.

**Figure 15:**
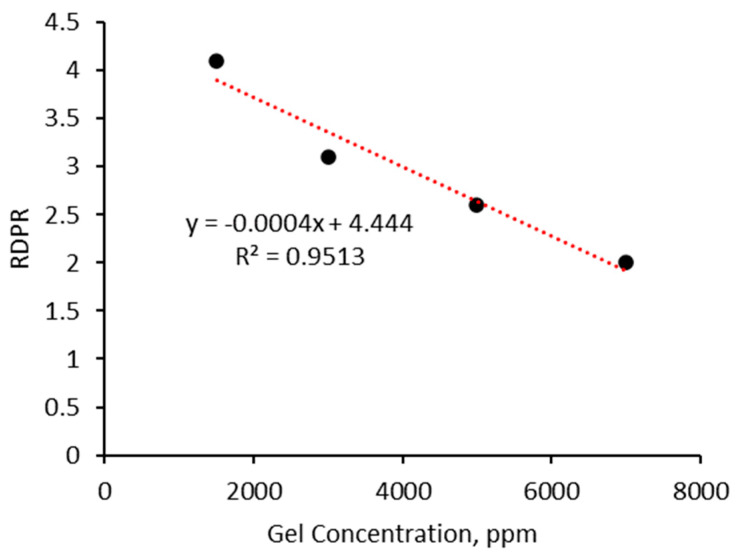
The relationship between gel concentration and RDPR reduction for CO_2_ and water.

The differential permeability reduction between water and CO_2_ highlights the gel’s selective plugging performance. In general, the gel tends to reduce the permeability of water more effectively than that of CO_2_, resulting in a higher residual resistance factor for water. This selective permeability reduction is beneficial for enhanced oil recovery processes, as it helps to control water production while allowing CO_2_ to maintain its mobility. Consequently, the gel’s ability to differentially reduce permeability enhances its overall effectiveness in improving the efficiency of CO_2_ oil recovery operations. Figure 15 shows the relationship between gel concentration and the disproportional permeability reduction ratio R_DPR_ for CO_2_ and water, as calculated using Equation (4). The trend line indicates a negative correlation, suggesting that as the gel concentration increases, the differential permeability reduction decreases. This implies that at lower gel concentrations, there is a higher selectivity in reducing water permeability compared with CO_2_ permeability.

In the context of Figure 14, this graph supports the observation that at higher gel concentrations (such as 5000 ppm and 7000 ppm), the plugging performance for CO_2_ improves due to reduced differential permeability. This means that while the gel effectively reduces the permeability of both water and CO_2_, the reduction is more balanced at higher concentrations, resulting in better overall plugging performance for CO_2_.

**Figure 16:**
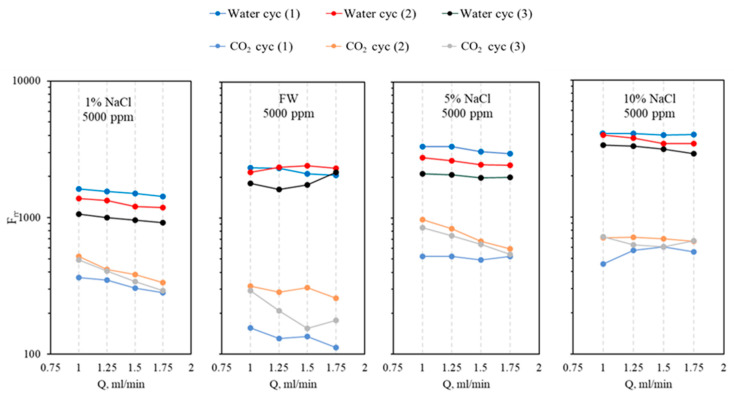
The residual resistance factor for both CO_2_ and water at different flow rates (1, 1.25, 1.5, and 1.75 mL/min) under varying salinity conditions with a constant gel concentration of 5000 ppm.

**Figure 17:**
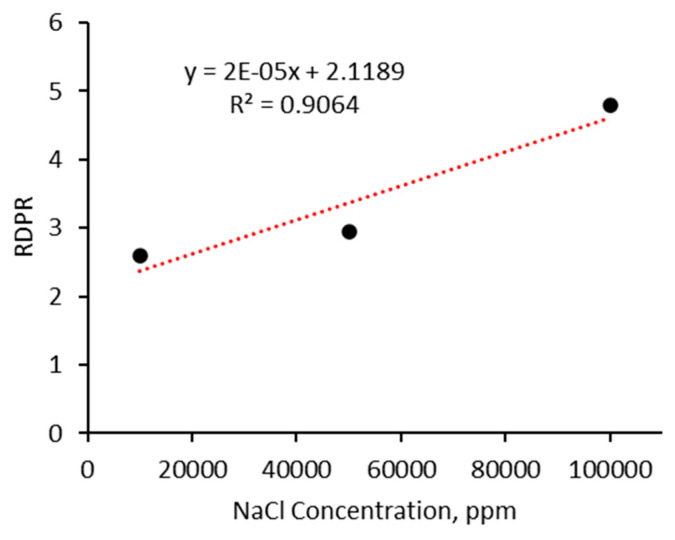
The relationship between NaCl concentration and RDPR for CO_2_ and water.

Figure 16 illustrates the effect of salinity on the residual resistance factor for both water and CO_2_ for the CO_2_-BRPPG at a concentration of 5000 ppm across four different salinity levels (1% NaCl, 5% NaCl, 10% NaCl, and formation water). In all cases, there is a consistent reduction in the F_rrw_ as the flow rate increases. However, in the formation water case, the F_rrw_ increases as the flow rate rises. One possible explanation is that the gel formed under formation water conditions is weaker, as shown by the data in this paper. The weaker gel structure allows the particles to move more easily at higher flow rates, leading to their accumulation at the outlet, which increases the blockage and raises the F_rrw_. In general, as salinity increases, the F_rrw_ for CO_2_ shows a more pronounced decrease with increasing flow rates, with the most significant reduction observed at 10% NaCl. This suggests that higher-salinity gels are more effective in selectively reducing CO_2_ permeability while maintaining water permeability, thereby creating greater differential permeability. This enhanced gel strength at higher salinity levels improves the gel’s ability to block CO_2_ flow, delaying CO_2_ breakthrough during flooding operations and ultimately enhancing oil recovery. This observation is further approved by plotting the relationship between NaCl concentration and RDPR, where increasing salinity leads to an increase in the permeability reduction, as shown in Figure 17. The high coefficient of determination (R^2^ = 0.9064) in the graph indicates that higher NaCl concentrations significantly enhance the gel’s effectiveness in reducing RDPR, supporting the observed trend in Figure 16.

## 3. Conclusions

The potential of the micro-sized CO_2_-BRPPG for permeability reduction in super-permeable channels was evaluated using sandpack models. Based on the experimental results obtained in this study, we can draw the following conclusions:

1. The micro-sized CO_2_-BRPPG demonstrates improved stability and effectiveness in high-salinity and varying-pH environments. At ambient conditions, the CO_2_-BRPPG can swell up to 76 times its original volume. This swelling ratio decreases with increasing salinity and remains relatively stable at low pH levels, demonstrating the gel’s excellent resistance to the acidic conditions resulting from CO_2_ flooding;
2. The micro-sized CO_2_-BRPPG exhibits shear-thinning behavior, with viscosity decreasing as the shear rate increases. This characteristic ensures that the gel can be easily injected under high-shear conditions near the wellbore but retains sufficient viscosity under low-shear conditions farther from wellbore to effectively block high-permeability channels;
3. The strength of the micro-sized CO_2_-BRPPG increases with higher salinity levels, with the storage modulus rising from 113 Pa at 1% NaCl to 145 Pa at 10% NaCl. This demonstrates that a reservoir with higher salinity improves the mechanical stability and rigidity of the gel, which enhances its effectiveness in blocking CO_2_ flow;
4. The injectivity of the micro-sized CO_2_-BRPPG can be controlled by adjusting the gel concentration and particle size. Low concentrations result in low injection pressure, which is suitable for deep injection treatments. For approximately equal-permeability media, highly swollen particles showed lower injection pressures than less swollen ones, likely due to their lower strength and better deformability;
5. The micro-sized CO_2_-BRPPG exhibits selective plugging performance, reducing water permeability more effectively than CO_2_. The ratio of F_rrw_ to F_rrg_ increases with higher brine concentrations and decreases with higher gel concentrations, indicating that gel strength significantly impacts this parameter.

## 4. Materials and Methods

### 4.1. Materials

**Figure 18:**
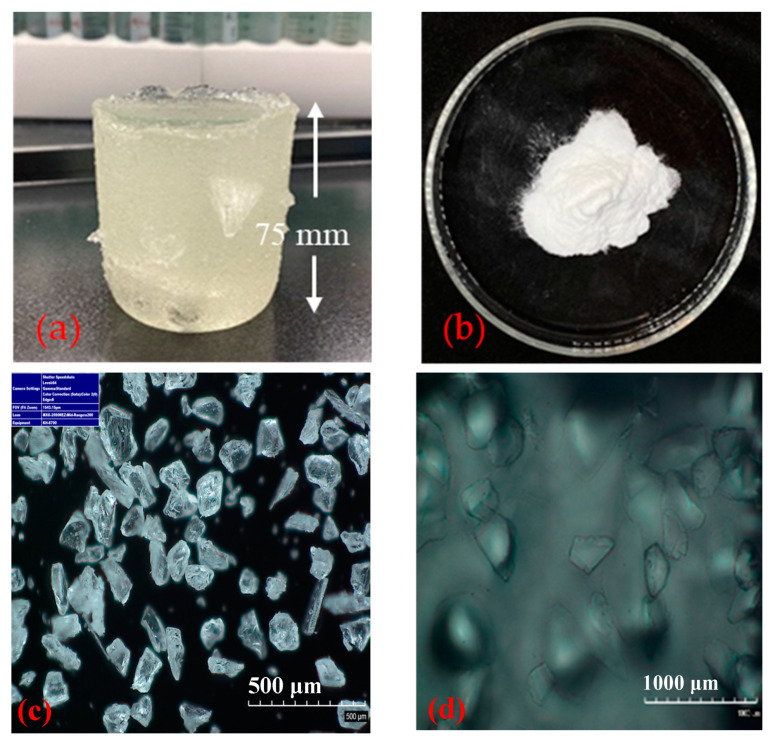
(**a**) shows the CO_2_-BRPPG bulk gel that formed after free-radical polymerization, (**b**) shows the CO_2_-BRPPG ground to a 170/230 mesh size, (**c**) shows a microscopic picture of dry CO_2_-BRPPG, and (**d**) shows a swelling particle gel.

**CO_2_-BRPPG**. The gel used in this study, based on the recipe in [35], is specifically synthesized with a high concentration of monomers that can withstand the acidic conditions created by CO_2_. Chemicals and reagents were primarily sourced from Sigma-Aldrich (St. Louis, MO, USA). The primary monomers acrylamide (AM) and 2-acrylamide-2-methylpropanesulfonic acid (AMPS) were copolymerized and crosslinked with L-lysine monohydrochloride (Lys-HCl), zirconium acetate, and N′-methylene bisacrylamide (MBA). The polymerization process was initiated by ammonium cerium (IV) nitrate (CAN) via free-radical polymerization in an aqueous solution. After the polymerization process, the gel was allowed to cool down at room temperature for 24 h and was dried in an oven at 65 °C for three days to reduce the water content. Subsequently, the dried gel was ground to a particle size of 170/230 (≈89 µm) (Figure 18).

**Brine and Gas.** In this study, four brines were used for swelling and carrying CO_2_-BRPPG: 1%, 5%, and 10% NaCl by weight, along with a synthesized formation water (FW) solution with a total dissolved solids (TDS) concentration of 57,000 ppm, as shown in Table 3. High-purity carbon dioxide and nitrogen (N_2_) were purchased from a local Airgas company (Rolla, MO, USA).

**Proppant Sand.** A quartz sand proppant of 20/30 mesh provided by Covia™ was used for all experiments. The relative density and sphericity of the quartz sand are 2.65 and 0.9, respectively.

### 4.2. Methods

**Particle Size Measurement and Swelling Ratio.** The particle size of fully swollen CO_2_-BRPPG prepared in brine with different salinity and pH values was measured using a HIROX KH-8700 digital microscope (Shibata Co., Ltd, Tokyo, Japan) equipped with low-range lenses at magnifications of 100× and 150×. A random sample of 100 fully swollen gel particles was selected, and their sizes were measured according to Feret’s diameter method, which involves measuring the distance between two parallel tangents on opposite sides of a particle, using image analysis software. The swelling ratio (*SR*) was determined using the volumetric method, as demonstrated in Equation (1) below:

(1) $$SR={\left(\frac{D_{t}}{d_{0}}\right)}^{3}$$

where *SR* is the swelling ratio, *d*_0_ is the initial average particle size of the gel particles (dry size), and *D_t_* is the average particle size of gel particles at time t during the swelling process [36].

**Rheology Test.** The rheology behavior of the CO_2_-BRPPG suspension was evaluated using the Brookfield (DV3T) Rheometer (Mokena, IL, USA) across a shear rate range of 5 to 1000 s^−1^. Samples with different concentrations, pH levels, and salinities were prepared, homogenized, and loaded into the rheometer without air bubbles. An appropriate spindle was selected, and the rheometer was set to control. The test gradually increased the shear rate, recording viscosity vs shear rate measurements.

The viscoelastic properties of the CO_2_-BRPPG were evaluated using a Haake MARS III rheometer (Thermo Scientific Inc., Waltham, MA, USA). The gel samples were prepared by removing the fully swollen particles from the gel particle suspension and sieving them for 24 h under ambient conditions to drain the excess water. The storage modulus (G’) and viscous modulus (G”) were measured using a P35Ti spindle, and the gap was set at 1 mm. The test was selected as the oscillation time-dependent experiments mode with a fixed frequency of 1 Hz and a controlled strain of 1%.

**Figure 19:**
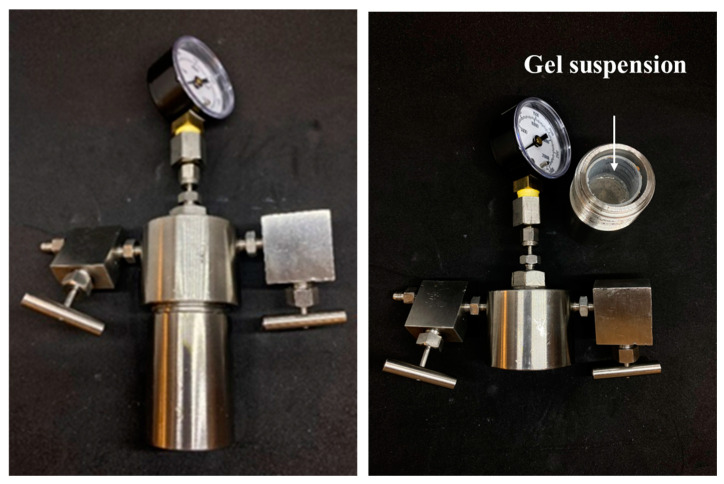
High-pressure vessel and loaded sample.

**Phase Stability Test.** The phase stability test is designed to evaluate changes in the properties (gel strength, particle size, swelling ratio, and gel morphology) of CO_2_-BRPPG after exposure to high-pressure CO_2_ conditions. A high-pressure vessel made of stainless steel is used in this test (Figure 19). The sample is placed in a container covered with filter paper to ensure that the gel suspension does not seep out of the vessel during pressure release. Once the sample is secured, CO_2_ gas is circulated to purge the system of air. After purging, the outlet valve is closed, allowing the pressure inside the vessel to increase to the designated level.

**Morphology Characterization.** To examine the morphology of the CO_2_-BRPPG before and after CO_2_ exposure using the Helios Hydra Cx SEM (Waltham, MA, USA) fully swollen gel particles were first collected and immersed in liquid nitrogen. The samples were then lyophilized for 48 h. Thin cross-sections of the lyophilized samples were then cut and sputter-coated with gold/palladium (Au/Pd). Finally, the coated samples were imaged using the SEM to analyze their morphology, and the magnification of the picture used was 200×.

**Figure 20:**
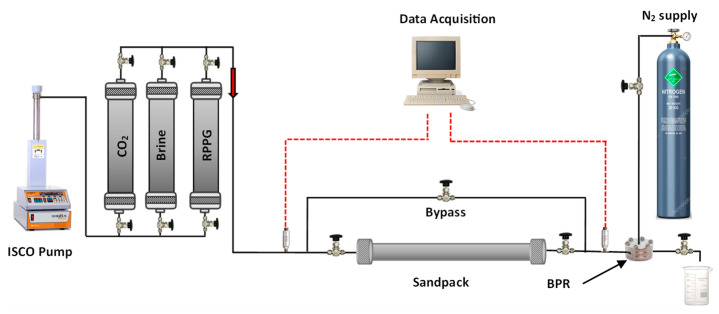
Schematic of the experimental setup for CO_2_/brine flooding.

**Injectivity and CO_2_ flooding test.** A flooding test was conducted to evaluate the injectivity and the plugging performance of the CO_2_-BRPPG. To simulate the reservoir’s high-permeability channel, a 2.5 × 20 cm sandpack was utilized. Figure 20 shows a schematic of the experimental sandpack setup. The basic information of each experiment is summarized in Table 4.

The sandpack was filled with weighted proppant with a 20/30 mesh size using the dry-packing method. After placing the sand, the tube was shaken in the vibrator machine to ensure tight packing. The porosity of the sandpack was found by calculating the bulk volume of the placed sand. The system was then vacuumed to remove the air and saturated with the designed brine solution for 24 h. For all experiments, the temperature was maintained at 45 °C, and CO_2_ was injected as a dense phase by keeping the back pressure at 850 psi. The general procedure used for the flow experiments was as follows:

1. The system’s initial permeabilities were evaluated by injecting brine and CO_2_ at different flow rates and calculating the permeability using the Darcy equation;
2. A suspension of the CO_2_-BRPPG (170/230 mesh) was prepared by immersing dry gel particles in brine until the equilibrium swelling ratio was reached. The swollen particles were then placed in a high-pressure magnetic stirrer accumulator to ensure a homogeneous suspension during the injection. The gel suspension was injected at a flow rate of 2 mL/min until gel breakthrough occurred and a stable injection pressure was achieved. The gel injectivity was evaluated by calculating the resistance factor (F_r_) during the injection process using Equation (2), where $\lambda_{w}$ is the water mobility ratio, $\lambda_{gel}$ is the gel mobility ratio, ${∆p}_{gel}$ is the differential pressure of the injected gel in psi, and ${∆p}_{ini-water}$ is the water differential pressure in psi;
  (2) $$F_{r}=\frac{\lambda_{w}}{\lambda_{gel}}=\frac{{∆p}_{gel}}{{∆p}_{ini-water}}$$
3. The system was shut in for three days, allowing the gel to recrosslink. The tubes and fittings connecting the sandpack with accumulators and the backpressure regulator were cleaned with water and air;
4. The CO_2_–water flooding process was carried out by injecting CO_2_ first to evaluate the gas breakthrough pressure in pressure stepwise mode. Three water-alternating-CO_2_ cycles were injected for the entire process. For both brine and CO_2_, the injection flow rates were 1, 1.25, 1.5, and 1.75 mL/min. The flow rate was changed once a stable injection pressure was achieved. The residual resistance factor (F_rr_) for both water and CO_2_ for each cycle was calculated using Equation (3), and the proportional permeability reduction was evaluated using Equation (4), where $K_{before}$ is the initial permeability of the system in mD, $K_{after}$ is the permeability after gel treatment in mD, and $R_{DPR}$ is the disproportional permeability reduction ratio.
  (3) $$F_{rr}={\left.\frac{K_{before}}{K_{after}}\right|}_{q}$$
  (4) $$R_{DPR}=\frac{F_{rrw}}{F_{rrg}}$$

## Figures and Tables

**Table 1:** The average equilibrium swelling ratio for the CO_2_-BRPPG was prepared using different salinity and pH solutions.

Sample #	Brine	pH	ESR (*V*/*V*)
1	1 NaCl%	≈7	76
2	5 NaCl%	≈7	52.4
3	10 NaCl%	≈7	60.9
4	FW	≈7	59.6
5	1 NaCl%	≈3.0	66
6	1 NaCl%	≈5.0	68
7	1 NaCl%	≈9.0	72
8	1 NaCl%	≈11.0	76

**Table 2:** Relationship between the CO_2_-BRPPG concentration and suspension salinity.

Exp No.	Gel Conc (ppm)	Brine	F_r_
1	1500	1 NaCl%	1318
2	3000	1 NaCl%	1707
3	5000	1 NaCl%	3977
4	7000	1 NaCl%	5116
5	5000	5 NaCl%	5750
6	5000	10 NaCl%	6214
7	5000	Formation water	2714

**Table 3:** Brine chemical composition of the formation water.

Composition	Na^+^/K^+^	Ca^2+^	Mg^2+^	Cl^−^	HCO_3_^−^	SO_4_^2−^	Total
Concentration (ppm)	20,043	2172	658	28,916	3063	2977	57,829

**Table 4:** Basic properties of the sandpack experiment.

Exp No.	Permeability (md)	Porosity (%)	PV (mL)	Gel Conc (ppm)	Brine
1	45	34.2	33	1500	1 NaCl%
2	49	32.8	35	3000	1 NaCl%
3	47	34.4	32.5	5000	1 NaCl%
4	49	34.7	32.5	7000	1 NaCl%
5	45	33.9	34	5000	5 NaCl%
6	48	34.4	35	5000	10 NaCl%
7	47	34.6	35	5000	Formation water

## Data Availability

All data and materials are available on request from the corresponding author. The data are not publicly available due to ongoing research using a part of the data.
